# Effects of Papaya Leaf Meal and Multi-Enzyme Supplementation on Growth Performance, Nutrient Digestibility, Carcass Traits, and Antioxidant Status in Arbor Acres Broiler Chickens

**DOI:** 10.3390/vetsci13030269

**Published:** 2026-03-14

**Authors:** Maha A. Abd El Latif, Ahmed A. A. Abdel-Wareth

**Affiliations:** 1 Department of Animal and Poultry Production, Faculty of Agriculture, Minia University, Minya 61519, Egypt; maha.omr@mu.edu.eg; 2 Poultry Center, College of Agriculture, Food and Natural Resources, Prairie View A & M University, Prairie View, TX 77446, USA; 3 Department of Animal and Poultry Production, Faculty of Agriculture, Qena University, Qena 83523, Egypt

**Keywords:** papaya leaf meal, multi-enzyme supplementation, broiler chickens, growth performance, nutrient digestibility, lipid profile, antioxidant status

## Abstract

The present study investigated the impact of dietary papaya leaf meal (PLM) at three inclusion levels (0%, 6%, and 12%) with or without multi-enzyme supplementation (0.5 g/kg diet) on growth performance, nutrient digestibility, carcass characteristics, serum biochemistry, lipid profile, and antioxidant status in Arbor Acres broiler chickens. A total of 240 one-day-old chicks were allocated to six treatments in a 3 × 2 factorial arrangement for 42 days. Enzyme supplementation significantly improved body weight gain, feed conversion ratio, and nutrient efficiency (*p* < 0.001), while moderate PLM inclusion (6%) supported optimal performance. Digestibility of crude fiber and ether extract was enhanced by enzymes (*p* < 0.05), with a notable PLM × enzyme interaction for fiber digestibility. Carcass yield was unaffected, but enzyme supplementation increased dressing percentage and reduced abdominal fat. Serum biochemistry remained largely unchanged, except for elevated AST at 12% PLM (*p* < 0.01). Lipid profile improved with enzyme supplementation, reducing total cholesterol, triglycerides, and LDL while increasing HDL (*p* < 0.05). Enzyme supplementation significantly increased SOD and CAT activities (*p* < 0.001), whereas TAC responses were inconsistent across treatments. Lipid peroxidation (MDA) increased at 12% PLM, indicating a potential oxidative imbalance at higher inclusion levels. In conclusion, multi-enzyme supplementation was the primary driver of growth performance and nutrient utilization improvements, while moderate PLM inclusion (6%) exerted supportive and synergistic effects without compromising carcass traits.

## Simple Summary

Papaya leaves contain beneficial nutrients and natural antioxidants, but their use in poultry feed has been limited due to their fiber content. This study examined whether adding papaya leaf meal (PLM) to broiler diets, with or without a multi-enzyme supplement, could enhance growth and health. We found that including a moderate level of PLM (6%), especially when combined with enzymes, improved growth performance, feed efficiency, nutrient digestibility, and antioxidant status. Enzyme supplementation also supported better lipid metabolism by lowering cholesterol and triglycerides. Overall, using PLM with enzymes offers a natural way to support broiler performance and physiological well-being.

## 1. Introduction

The global poultry industry continues to face sustained pressure to maximize productivity and bird health while reducing dependence on antibiotic growth promoters (AGPs). Increasing regulatory restrictions and heightened consumer awareness regarding antimicrobial resistance have accelerated the adoption of non-antibiotic nutritional strategies that preserve performance without compromising food safety. Within this context, phytogenic feed additives, defined as plant-derived bioactive compounds incorporated into animal diets to improve performance, health, and product quality, and exogenous enzyme complexes have emerged as practical, evidence-based tools to enhance nutrient utilization, growth performance, and metabolic efficiency in broiler production systems [1]. Accumulating experimental and field-based evidence demonstrates that diets incorporating botanical ingredients, combined with tailored enzyme blends, can improve body weight gain, feed conversion ratio (FCR), and overall production efficiency, providing a robust framework for AGP-free broiler nutrition [1,2].

Papaya (*Carica papaya*) leaf meal (PLM) is a representative phytogenic feed additive, offering a rich profile of proteolytic enzymes, particularly papain, as well as diverse bioactive compounds such as flavonoids, alkaloids, saponins, and phenolics. These constituents have been associated with enhanced protein digestion, antioxidant activity, immunomodulation, and hypocholesterolemic effects in poultry [3,4,5]. Previous studies using inclusion levels of approximately 1–9% PLM have reported improvements in growth performance and feed conversion ratio, suggesting that papain may augment endogenous proteolysis, while polyphenolic compounds contribute to gut integrity and metabolic homeostasis [1,3,5]. However, PLM is also characterized by relatively high concentrations of structural carbohydrates, particularly non-starch polysaccharides (NSPs), which can increase digesta viscosity, encapsulate nutrients, and limit their accessibility to endogenous enzymes [1,2]. In addition, antinutritional factors such as saponins and tannins may further constrain nutrient utilization at higher inclusion levels [3,6]. Consequently, the application of exogenous multi-enzymes capable of hydrolyzing NSP fractions may enhance nutrient release and mitigate fiber-related constraints, providing a mechanistic basis for evaluating the interactive effects of PLM and enzyme supplementation in a factorial design.

Exogenous multi-enzyme complexes—typically comprising carbohydrases (e.g., xylanase, β-glucanase, pectinase, mannanase), amylases, proteases, and, in some formulations, phytase—enhance digestive efficiency by degrading NSPs, reducing intestinal viscosity, releasing encapsulated nutrients, and complementing endogenous digestive capacity [1,2,4]. These mechanisms collectively improve the availability of starch, protein, fat, and minerals, thereby enhancing growth performance and feed efficiency. Recent broiler studies indicate that enzyme supplementation alone can significantly increase the apparent digestibility of dry matter (DM), crude protein (CP), ether extract (EE), and ash, and that combining enzymes with PLM may yield additive or synergistic improvements in nutrient utilization [1,4].

Processing strategies further influence the nutritional value and applicability of PLM in broiler diets. Techniques such as fermentation and acidification have been explored to reduce antinutritional factors, modify fiber characteristics, and improve palatability, thereby expanding the safe inclusion range of PLM [3,4,6]. Fermented PLM at inclusion levels up to approximately 12% has been shown to enhance feed intake, body weight gain, and FCR compared with unprocessed leaf meal, highlighting the importance of pre-treatment when higher dietary levels are targeted [3]. Acidified papaya leaf and seed mixtures have also been evaluated, with variable performance outcomes; yet, collectively, these approaches underscore the feasibility of biochemical conditioning to optimize PLM functionality in practical broiler rations [4,6].

Beyond growth performance, PLM and enzyme supplementation have been shown to influence metabolic and health-related indices in broilers. Favorable alterations in serum lipid profiles, including reductions in total cholesterol and low-density lipoprotein (LDL) cholesterol and increases in high-density lipoprotein (HDL) cholesterol, have been reported with PLM inclusion, potentially mediated by saponin-induced reductions in intestinal cholesterol absorption and modulation of hepatic lipid metabolism [1,6]. Enzyme supplementation may further complement these effects by enhancing lipid digestion efficiency and bile acid utilization, thereby improving lipid transport dynamics and reducing circulating triglycerides and LDL concentrations [1,2,4]. In addition to lipid metabolism, plant-derived bioactive compounds have been increasingly recognized for their capacity to modulate intestinal fermentation patterns and oxidative status. Recent evidence indicates that phytogenic additives can enhance antioxidant enzyme activity, reduce lipid peroxidation, and shift microbial fermentation toward more favorable metabolite profiles, thereby improving gut environment and systemic metabolic stability [7,8]. Such findings provide biological plausibility for the hypothesis that PLM bioactives, particularly polyphenols and flavonoids, may influence both oxidative balance and intestinal metabolic processes. When combined with exogenous enzymes that improve substrate availability and nutrient release, these bioactives may exert synergistic effects on fermentation dynamics and antioxidant defense systems, thereby enhancing metabolic resilience [2,4,7,8]. Collectively, these responses suggest that PLM–enzyme combinations may support cardiometabolic and oxidative stability, outcomes increasingly relevant in antibiotic-free intensive broiler production systems focused on robustness and product quality [7,8].

Antioxidant status represents an additional dimension through which PLM–enzyme strategies may exert beneficial effects. The polyphenolic compounds present in PLM contribute directly to total antioxidant capacity (TAC), while enhanced nutrient release and mineral bioavailability mediated by enzyme supplementation may support the activity of endogenous antioxidant enzymes, such as superoxide dismutase and catalase [1,2,3,4,5]. Nonetheless, excessive PLM inclusion may increase the oxidative burden due to elevated fiber and antinutritional factor content, underscoring the importance of optimized inclusion rates and enzyme supplementation. Evidence suggests that low to moderate PLM levels improve productivity without adverse effects on serum biochemistry, whereas higher inclusion levels may require processing or targeted enzyme supplementation to maintain redox and metabolic balance [3,4,5,6]. Despite growing interest in PLM and enzyme supplementation, critical knowledge gaps remain regarding the dose–response effects of graded PLM inclusion and its interaction with comprehensive enzyme blends under commercial broiler conditions. Previous studies have frequently evaluated single PLM inclusion levels [1,3,5] or focused on processing methods in isolation [4], rather than systematically assessing factorial combinations of PLM and multi-enzyme supplementation across production phases. These studies primarily assessed PLM as a single dietary factor, without consideration of multi-enzyme supplementation.

The current 3 × 2 factorial design was therefore employed to evaluate potential synergistic effects of PLM and exogenous enzyme complexes on nutrient utilization, growth performance, and metabolic efficiency in broilers. Accordingly, the objective of the present study was to evaluate the effects of graded PLM inclusion (0%, 6%, and 12%), with or without a multi-enzyme complex, on growth performance, nutrient digestibility, carcass characteristics, serum biochemistry, lipid profile, and antioxidant status of Arbor Acres broilers over a 42-day production cycle. This study aimed to determine whether multi-enzyme supplementation could enhance nutrient utilization and metabolic responses at moderate PLM inclusion and mitigate potential negative effects associated with higher PLM levels, thereby providing practical guidance for the enzyme-assisted use of papaya leaf meal in antibiotic-free broiler nutrition programs.

## 2. Materials and Methods

### 2.1. Animals, Diets, and Experimental Design

A total of 240 one-day-old straight-run Arbor Acres broiler chicks were obtained from a commercial hatchery and randomly assigned to six dietary treatments using a randomized complete block design in a 3 × 2 factorial arrangement. Birds were initially stratified according to body weight at placement and then randomly allocated to treatments within blocks to minimize variation among experimental units. The experimental factors consisted of three levels of papaya leaf meal (PLM) inclusion (0%, 6%, and 12%) and two levels of multi-enzyme supplementation (0 or 0.5 g/kg diet). The multi-enzyme product (Fra-Zyme^®^; Kemin Industries, Inc., Des Moines, IA, USA) was incorporated into the diets of the supplemented groups at a rate of 0.5 g/kg.

Each dietary treatment comprised four replicates, with 10 birds allocated per replicate pen, resulting in 40 birds per treatment. The experimental period lasted 42 days and was divided into a starter phase (1–21 days) and a grower phase (22–42 days). Birds were weighed individually at placement to ensure uniform initial body weight distribution among treatments.

Mash diets were formulated to meet or exceed the nutrient requirements recommended for Arbor Acres broilers during each feeding phase (Table 1). All diets were isoenergetic and isonitrogenous within each phase. The enzyme activity per gram of Fra-Zyme^®^ was declared as follows: xylanase (*Trichoderma reesei*), 16,000 BXU; β-glucanase (*Trichoderma reesei*), 2400 BU; pectinase (*Aspergillus niger*), 210 U; α-amylase (*Bacillus subtilis*), 21,000 IU; mannanase (*Trichoderma reesei*), 3000 MNU; and protease (*Bacillus subtilis*), 600 U.

### 2.2. Housing and Management

Birds were housed in floor pens under deep-litter conditions at the Poultry Research Unit, Faculty of Agriculture, Minia University. Each pen (180 × 100 × 80 cm) was bedded with clean wood shavings. Feed and water were provided *ad libitum* throughout the experimental period using manual feeders and drinkers.

Environmental temperature was maintained at approximately 34 °C during the first week of age and gradually reduced by 2–3 °C per week to reach 24 °C by the fourth week, where it was maintained until the end of the trial. Lighting, ventilation, and general husbandry practices followed standard commercial broiler management recommendations. Birds were monitored daily for health status and mortality.

All experimental procedures involving animals were conducted in accordance with institutional and national guidelines for the care and use of laboratory animals. The study protocol was reviewed and approved by the Institutional Animal Care and Use Committee of Minia University (Approval No. MU/FA027/07/25). All animal handling and experimental procedures followed the recommendations outlined in The Guide for the Care and Use of Laboratory Animals in Scientific Investigations.

### 2.3. Preparation of Papaya Leaf Meal

Fresh papaya leaves (*Carica papaya*) were collected from the Horticulture Department, Faculty of Agriculture, Minia University. The leaves were washed thoroughly with tap water to remove dirt and debris, then shade-dried at ambient temperature until a constant weight was achieved. Dried leaves were ground using a hammer mill to obtain a fine powder and stored in airtight containers until diet formulation.

The proximate composition of papaya leaf meal, including moisture, ash, crude protein, crude fiber, ether extract, and nitrogen-free extract, was determined according to standard methods described by the Association of Official Analytical Chemists [9].

### 2.4. Growth Performance

Body weight and feed intake were recorded on a pen basis at 1, 21, and 42 days of age. Body weight gain (BWG), feed intake (FI), and feed conversion ratio (FCR) were calculated for the starter (0–21 days), grower (22–42 days), and overall (0–42 days) periods. Mortality was recorded daily, and performance data were adjusted for mortality when applicable. Protein efficiency ratio (PER) and energy efficiency ratio (EER) were calculated using body weight gain and cumulative nutrient intake during the experimental period. PER was calculated as the ratio of total body weight gain to the total protein consumed by each bird. EER was calculated as the ratio of total body weight gain to the cumulative metabolizable energy (ME) intake. Protein intake was obtained by multiplying total feed intake by the dietary crude protein fraction, while ME intake was calculated by multiplying total feed intake by the dietary ME value.

### 2.5. Nutrient Digestibility

Digestibility trials were conducted from day 35 to day 42 using individual metabolic cages (70 × 60 × 40 cm) equipped with nipple drinkers and manual feeders. One bird from each replicate pen was randomly selected and transferred to an individual metabolic cage, resulting in four birds per treatment. During the collection period, birds were housed individually, and each bird (metabolic cage) was considered the experimental unit for digestibility measurements. Following a 3-day adaptation period, total excreta were quantitatively collected for four consecutive days. Collected excreta were pooled per bird, oven-dried at 60 °C for 48 h, weighed, and finely ground for chemical analysis. Representative samples of feed and excreta were analyzed using AOAC International methods [9] for moisture (AOAC 930.15), ash (AOAC 942.05), crude protein (AOAC 984.13), ether extract (AOAC 954.02), and crude fiber (AOAC 978.10). Apparent nutrient digestibility coefficients were calculated using standard equations.

### 2.6. Carcass Traits

At 42 days of age, 16 birds per treatment (four birds per replicate) were randomly selected following a 12 h feed withdrawal and individually weighed. Birds were slaughtered according to institutional animal care and poultry processing guidelines, including humane cervical dislocation, complete bleeding, defeathering, evisceration, and standardized separation of carcass components. Carcass weight and the weights of other components were then recorded.

Carcass yield, dressing percentage, and relative organ weights (heart, spleen, empty gizzard, and abdominal fat) were expressed as percentages of live body weight. Dressing percentage was calculated as follows:

$$\mathrm{Dressing}\;\mathrm{percentage}=\frac{\mathrm{carcass}\;\mathrm{weight}\;}{\mathrm{live}\;\mathrm{body}\;\mathrm{weight}}\times 100$$

### 2.7. Blood Biochemical and Antioxidant Assays

At 42 days of age, blood samples were collected from the brachial (wing) vein of sixteen birds per treatment group using sterile, non-heparinized vacutainer tubes. Immediately after collection, blood samples were left undisturbed to clot at room temperature (approximately 22–25 °C) for 30–40 min. Clotted samples were then centrifuged at 3000× *g* for 15 min to obtain clear serum. The resulting serum fraction was carefully transferred into labeled microtubes and stored at −20 °C until biochemical and antioxidant analyses were performed.

Serum antioxidant status was evaluated using well-established colorimetric procedures. Total antioxidant capacity (TAC) was measured according to the method of Koracevic et al. [10], while lipid peroxidation was quantified by determining malondialdehyde (MDA) concentration using the thiobarbituric acid reactive substances (TBARS) assay described by Ohkawa et al. [11]. Enzymatic antioxidant activities of superoxide dismutase (SOD) and catalase (CAT) were assessed following the methods of Nishikimi et al. [12] and Aebi [13], respectively.

Serum biochemical constituents, including total protein, albumin, glucose, alanine aminotransferase (ALT), aspartate aminotransferase (AST), triglycerides, total cholesterol, high-density lipoprotein (HDL), and low-density lipoprotein (LDL), were determined using commercially available diagnostic kits (Bio-Diagnostic, Cairo, Egypt), following the manufacturer’s instructions. Serum globulin concentration was calculated by subtracting albumin from total serum protein (Globulin = Total Protein − Albumin).

### 2.8. Statistical Analysis

Data were analyzed using two-way analysis of variance (ANOVA) to determine the effects of PLM inclusion level, enzyme supplementation, and their interaction (PLM × enzyme) using SAS software, version 9.2 [14]. Prior to conducting ANOVA, data were tested for normal distribution using the Shapiro–Wilk test and for homogeneity of variances using Levene’s test to ensure compliance with model assumptions. Results were expressed as mean ± standard error of the mean (SEM). When significant treatment effects were detected, Duncan’s multiple-range test was used for pairwise comparisons of means [15]. Statistical significance was declared at *p* < 0.05.

## 3. Results

### 3.1. Growth Performance

The effects of dietary PLM inclusion and multi-enzyme supplementation on broiler growth performance are presented in Table 2. Initial body weight at day 0 did not differ among treatments (*p* > 0.05), indicating a uniform starting population.

On day 21, birds receiving enzyme-supplemented diets exhibited significantly greater body weight compared with non-supplemented groups (*p* < 0.001), whereas PLM inclusion level had no significant effect at this stage (*p* = 0.124). By day 42, both PLM level and enzyme supplementation significantly influenced final body weight (*p* < 0.001), with the highest values observed in birds fed diets containing 6% PLM supplemented with enzymes (Table 2).

Body weight gain during the starter phase (1–21 d) was significantly improved by enzyme supplementation (*p* < 0.001), while PLM inclusion had no detectable effect (*p* = 0.128). During the grower phase (22–42 d), both PLM level (*p* = 0.025) and enzyme supplementation (*p* < 0.001) significantly affected weight gain, with birds receiving 6% PLM combined with enzyme supplementation achieving the greatest gains. Similarly, overall weight gain (1–42 d) was primarily enhanced by enzyme supplementation (*p* < 0.001), whereas PLM inclusion exerted a moderate but significant effect (*p* = 0.001).

Feed intake was not significantly influenced by PLM inclusion across any period (*p* > 0.05). Enzyme supplementation increased feed intake during the starter phase (*p* < 0.001) but slightly reduced intake during the grower phase (*p* = 0.0001), resulting in no significant difference in cumulative feed intake over the entire experimental period (*p* = 0.498).

The interaction between PLM level and enzyme supplementation was not significant for most growth parameters (*p* > 0.05); however, birds fed 6% PLM with enzyme supplementation consistently exhibited numerically superior growth performance compared with other treatments.

### 3.2. Feed, Protein, and Energy Efficiency

The effects of PLM inclusion and enzyme supplementation on FCR, protein efficiency ratio (PER), and energy efficiency ratio (EER) are summarized in Table 3. During the starter phase (1–21 d), FCR was significantly improved by enzyme supplementation (*p* = 0.0003), while PLM level had no significant effect (*p* = 0.217). Birds receiving enzyme-supplemented diets showed lower FCR values compared with non-supplemented birds (1.60 vs. 1.75). A similar response was observed over the overall period (1–42 d), in which enzyme supplementation significantly reduced FCR (*p* = 0.0002). PLM inclusion exerted a moderate effect on overall FCR (*p* = 0.007), with the lowest values recorded at 6% PLM.

PER during the starter phase was not significantly influenced by PLM level or enzyme supplementation (*p* > 0.05). During the grower phase, enzyme supplementation tended to improve PER (*p* = 0.078), whereas the interaction effect was not significant (*p* = 0.510). Over the entire experimental period, enzyme supplementation significantly increased PER (*p* = 0.003), with the highest value observed in birds fed 6% PLM with enzymes.

EER followed a similar pattern. Enzyme supplementation significantly enhanced EER during the starter and overall periods (*p* = 0.0002), while PLM inclusion had a minor effect during the overall period (*p* = 0.026). No significant PLM × enzyme interactions were detected for any efficiency parameter (*p* > 0.05).

Overall, enzyme supplementation markedly improved feed utilization and nutrient efficiency, while moderate PLM inclusion (6%) was associated with more favorable efficiency indices compared with 0% or 12% inclusion.

### 3.3. Nutrient Digestibility

The effects of PLM inclusion and enzyme supplementation on nutrient digestibility are presented in Table 4. DM and OM were not significantly affected by PLM level or enzyme supplementation (*p* > 0.05), although enzyme-supplemented diets showed numerically higher values.

CP digestibility was not influenced by PLM inclusion (*p* = 0.943) but tended to increase with enzyme supplementation (*p* = 0.074. In contrast, CF digestibility was significantly affected by enzyme supplementation (*p* = 0.005) and by the PLM × enzyme interaction (*p* = 0.013). Birds receiving enzyme-supplemented diets exhibited higher CF digestibility, with the greatest values observed at higher PLM inclusion levels, suggesting that enzyme responses varied with dietary fiber level.

EE digestibility was significantly improved by enzyme supplementation (*p* = 0.046), while PLM level had no significant effect (*p* = 0.305). Digestibility of nitrogen-free extract (NFE) was not affected by either factor (*p* > 0.05).

Overall, enzyme supplementation enhanced fiber and fat digestibility, and the significant interaction for CF digestibility indicates that enzyme supplementation was particularly beneficial at higher PLM inclusion levels.

### 3.4. Carcass Yield and Organ Weights

The effects of PLM inclusion and enzyme supplementation on carcass characteristics are shown in Table 5. Live body weight and carcass weight were not significantly affected by either PLM level or enzyme supplementation (*p* > 0.05). In contrast, dressing percentage was significantly higher with enzyme supplementation (0.5 g/kg; 79.02%) than with non-supplemented diets (0 g/kg; 76.66%; *p* = 0.025), while PLM had no significant effect (*p* = 0.471).

Relative weights of the liver, gizzard, and heart were not significantly influenced by PLM inclusion or enzyme supplementation (*p* > 0.05). Abdominal fat increased with higher PLM inclusion (*p* = 0.364), with the highest fat percentage observed in birds fed 6% PLM, while enzyme supplementation tended to reduce fat deposition (*p* = 0.192). Giblet percentage was significantly affected by both PLM level (*p* = 0.023) and enzyme supplementation (*p* = 0.034), with the highest values observed in birds who fed 6% PLM and lower values in enzyme-supplemented groups. A significant PLM × enzyme interaction was detected for liver percentage (*p* = 0.007), indicating that enzyme supplementation moderated the increase in liver weight associated with higher PLM inclusion. No other interaction effects were observed.

### 3.5. Serum Biochemistry

Serum biochemical responses to PLM inclusion and enzyme supplementation are presented in Table 6. Concentrations of total protein, albumin, and globulin were not significantly affected by either factor (*p* > 0.05), although enzyme-supplemented birds exhibited numerically higher total protein and globulin values.

Serum glucose concentration was not influenced by PLM level (*p* = 0.813) or enzyme supplementation (*p* = 0.150). ALT activity tended to decrease with enzyme supplementation (*p* = 0.057), while PLM inclusion had no significant effect (*p* = 0.135). AST activity was significantly affected by PLM level (*p* = 0.001), with birds receiving 12% PLM exhibiting the highest values. Enzyme supplementation did not influence AST activity (*p* = 0.225), and no significant PLM × enzyme interactions were detected for serum biochemical indices. Enzyme supplementation did not influence AST activity (*p* = 0.225), and no PLM × enzyme interactions were detected for serum biochemical indices.

### 3.6. Serum Lipid Profile

The effects of PLM inclusion and enzyme supplementation on serum lipid parameters are summarized in Table 7. Total cholesterol concentration was significantly affected by both PLM level (*p* = 0.022) and enzyme supplementation (*p* = 0.009). Birds fed 6% PLM exhibited the highest cholesterol values, whereas enzyme supplementation significantly reduced total cholesterol compared with non-supplemented diets.

Triglyceride concentration tended to decrease with enzyme supplementation (*p* = 0.014) and was marginally influenced by PLM level (*p* = 0.066), with the lowest values observed in birds fed 12% PLM. HDL concentration was significantly increased by enzyme supplementation (*p* = 0.031), while PLM level had no effect (*p* = 0.374). LDL concentration was markedly reduced by enzyme supplementation (*p* = 0.003), whereas PLM inclusion had no significant effect (*p* = 0.343). Overall, enzyme supplementation favorably modulated lipid metabolism by reducing total cholesterol, triglycerides, and LDL while increasing HDL concentration.

### 3.7. Antioxidant Profile

Serum antioxidant responses are presented in Table 8. TAC was not significantly affected by PLM inclusion (*p* = 0.943) but tended to increase with enzyme supplementation (*p* = 0.088). MDA concentration was significantly influenced by PLM level (*p* = 0.045) and enzyme supplementation (*p* = 0.0002), with the highest values observed in birds fed 12% PLM. Superoxide dismutase (SOD) activity was significantly affected by PLM level, enzyme supplementation, and their interaction (*p* < 0.01), with the highest activity recorded in birds fed 12% PLM with enzyme supplementation. CAT activity was significantly increased by enzyme supplementation (*p* < 0.0001), and a significant PLM × enzyme interaction (*p* = 0.019) indicated greater enhancement of CAT activity at higher PLM inclusion levels.

Overall, enzyme supplementation markedly enhanced antioxidant enzyme activities and TAC, whereas higher PLM inclusion (12%) was associated with increased lipid peroxidation, indicating elevated oxidative challenge at higher inclusion levels.

## 4. Discussion

The present study evaluated the interactive effects of graded inclusion levels of papaya leaf meal (PLM; 0, 6, and 12%) and a multi-enzyme complex on growth performance, nutrient digestibility, carcass traits, lipid metabolism, antioxidant status, and selected serum biochemical indices of Arbor Acres broilers. The results demonstrate that enzyme supplementation enhanced growth efficiency, nutrient utilization, carcass yield, lipid profile, and antioxidant defense, whereas high PLM inclusion (12%) induced mild oxidative and metabolic stress. These findings are consistent with previous reports indicating that papaya (*pawpaw*) leaf-based diets can support broiler productivity when inclusion levels are properly optimized and, where necessary, supported with processing or dietary strategies [1,2,3,4,5,16,17].

The improvement in body weight gain and feed conversion ratio observed with enzyme supplementation can be mechanistically attributed to enhanced degradation of NSPs and improved nutrient accessibility [1,2,4,18]. Carbohydrases, including xylanase, β-glucanase, mannanase, and pectinase, hydrolyze structural carbohydrates, reduce digesta viscosity, and release encapsulated nutrients, thereby facilitating the action of endogenous digestive enzymes and promoting nutrient absorption and metabolizable energy utilization [19,20]. Complementary inclusion of α-amylase and protease further supports starch and protein hydrolysis, increasing amino acid availability for muscle deposition and tissue accretion [21,22]. These effects align with established NSP–enzyme interaction theory, which emphasizes that the efficacy of exogenous enzymes depends on the structural complexity and physicochemical properties of dietary fibers. In diets containing papaya leaf meal, rigid plant cell walls and high fiber content may impede nutrient access by encapsulating proteins, lipids, and starch, highlighting the critical role of multi-enzyme supplementation in improving nutrient utilization from both conventional feedstuffs and fibrous botanical ingredients. Exogenous enzyme supplementation becomes particularly important in these contexts, as it selectively degrades hemicellulose, pectin, and other NSPs, improving nutrient bioavailability. The meta-analysis by Bakare et al. [2] highlighted that leaf meal inclusion in broiler diets produces variable growth responses depending on fiber content and diet formulation, supporting the concept that enzyme supplementation is strategically beneficial in fibrous, phytogenic diets. Similarly, previous studies demonstrated that moderate papaya leaf supplementation improved growth performance and feed efficiency, likely due to the combination of bioactive compounds and improved nutrient accessibility [1,3,5].

The pattern observed in the present study, higher feed intake during the starter phase followed by improved feed efficiency in the grower–finisher phase, suggests that enzyme supplementation supports early gut development, potentially by reducing digesta viscosity and alleviating the mechanical and chemical barriers imposed by NSPs. Improved early gastrointestinal adaptation enhances digestive enzyme activity, nutrient absorption, and microbial balance, ultimately translating into superior growth and more efficient feed utilization throughout the production cycle. This dynamic is particularly relevant for broilers receiving fibrous botanical ingredients such as PLM, where the structural carbohydrate content can limit performance unless enzymatic action mitigates it.

Furthermore, enzyme supplementation may have indirect effects on gut health and oxidative balance. By promoting more complete nutrient digestion and reducing substrate availability for potentially pathogenic microbes, enzymes can lower gut inflammation and oxidative stress, thereby further supporting growth performance and metabolic efficiency. This mechanistic link between nutrient digestibility, gut microbiota modulation, and systemic health has been suggested in prior studies evaluating pawpaw leaf-based diets in broilers [1,4].

Overall, the present results indicate that multi-enzyme complexes can effectively counteract the structural limitations of PLM, improving growth performance, feed efficiency, and nutrient utilization, particularly when leaf meal inclusion levels are moderate and compatible with enzyme action. These findings underscore the importance of integrating exogenous enzymes into diets containing fibrous botanical ingredients to maximize their functional benefits while minimizing negative effects on metabolism and oxidative balance.

Papaya leaves contain papain, alkaloids, flavonoids, phenolic compounds, carotenoids, and vitamins that exert antioxidant, antimicrobial, and hypolipidemic effects. At 6% inclusion, PLM likely delivers these bioactive compounds at physiologically beneficial concentrations without imposing excessive fiber or antinutritional factors. Studies by Oloruntola et al. [15] reported that pawpaw leaf and seed meal supplementation maintained stable blood indices and induced beneficial histological changes in the liver and testis, supporting the notion that moderate inclusion can enhance systemic health in broilers. Importantly, our results show that the most favorable growth performance, feed efficiency, and oxidative status were achieved when moderate PLM inclusion was combined with enzyme supplementation, rather than with PLM alone. Enzymes enhance fiber degradation and nutrient release, allowing the bioactive compounds in PLM to exert their full physiological benefits. Similarly, Hasanah et al. [5] observed improved productivity with controlled papaya leaf meal inclusion, while Berihun [6] reported enhanced production performance when papaya leaves were incorporated in broiler diets, particularly when combined with complementary protein sources or enzyme supplementation. Collectively, these findings highlight that moderate PLM inclusion promotes growth and metabolic stability most effectively when synergistically paired with enzymatic support.

However, increasing PLM to 12% likely elevated dietary fiber, tannins, and saponins to levels that impose physiological stress. The rise in serum AST and MDA observed at this inclusion level indicates increased metabolic turnover and lipid peroxidation. Comparable findings have been reported when papaya leaf meal is included at higher levels without adequate processing or dietary balancing [17]. The meta-analysis by Bakare et al. [2] also concluded that excessive inclusion of leaf meal may impair performance due to a structural carbohydrate burden and the presence of antinutritional compounds.

The significant improvement in crude fiber and ether extract digestibility with enzyme supplementation reflects effective NSP degradation and improved nutrient release. By breaking down hemicellulose and pectic substances, carbohydrases reduce nutrient encapsulation and enhance lipid emulsification and micelle formation in the small intestine [18,19]. The significant PLM × enzyme interaction observed for crude fiber digestibility suggests that the efficacy of exogenous enzymes depends on the dietary structural carbohydrate load. Higher PLM inclusion increased the dietary fiber fraction, likely providing greater substrate availability for enzyme-mediated hydrolysis of non-starch polysaccharides, thereby improving fiber degradation. Similar structural diet–enzyme interactions have been reported in other livestock systems, where dietary fiber manipulation alters fermentation dynamics and nutrient utilization efficiency [23]. Although species differ in digestive physiology, these findings support the broader concept that structural feed composition can influence the functional efficacy of feed enzymes.

Notably, Nababan et al. [3] demonstrated that fermentation of papaya leaf flour improved broiler performance, indicating that structural modification of leaf fiber enhances nutrient availability. Similarly, Sugiharto et al. [4] showed that acidification of papaya leaf and seed meal improved growth and carcass traits, suggesting that reducing antinutritional factors and modifying fiber structure enhances digestibility. These findings support the mechanistic role of enzymes in degrading structural barriers and improving nutrient extraction in PLM-containing diets.

The modest changes in crude protein digestibility observed in the present study suggest that NSP-associated encapsulation, rather than intrinsic protein digestibility, was the primary limitation. Protease supplementation may have improved amino acid availability for growth without markedly altering apparent digestibility coefficients during the balance period.

Enzyme supplementation significantly improved dressing percentage and reduced abdominal fat deposition, indicating more efficient nutrient partitioning toward lean tissue. Mechanistically, enhanced digestibility increases the availability of metabolizable energy and amino acids, which supports muscle protein deposition rather than energy storage as fat [16,20,21]. Improved lipid metabolism, reflected by decreased LDL and increased HDL, further contributes to lean tissue accretion. The combination of 6% PLM with enzymes appears to synergistically optimize nutrient partitioning by enhancing digestion and providing bioactive compounds that support metabolic regulation. This shift toward lean tissue is particularly beneficial in broiler production, where feed efficiency and carcass quality are critical economic and quality indicators.

Previous studies have also reported improvements in carcass characteristics when papaya leaf meal is processed or included at optimized levels. For example, Sugiharto et al. [4] observed higher carcass yield in broilers fed acidified papaya leaf and seed meal, and Nababan et al. [3] demonstrated enhanced growth performance and tissue accretion following fermentation of papaya leaf flour. These findings suggest that structural modification of leaf meals through either enzymatic degradation or fermentation/acidification can improve nutrient availability, support lean tissue growth, and reduce excessive fat deposition [3,24]. The present results align with these observations, showing that multi-enzyme supplementation mitigates the structural limitations of PLM and enhances carcass yield even at higher inclusion levels.

The observed interaction effect on liver relative weight further suggests that enzyme supplementation may reduce hepatic metabolic burden at high PLM inclusion levels. By improving lipid digestion and reducing the amount of undigested fiber and phytochemicals entering the liver for processing, enzymes may lower detoxification demands and support normal hepatic function, thereby improving overall nutrient utilization and metabolic efficiency.

Enzyme supplementation favorably modulated serum lipid metabolism, suggesting improved lipid digestion, absorption, and systemic transport. Mechanistically, exogenous enzymes degrade NSPs and reduce intestinal viscosity, thereby facilitating bile acid recycling, improving micelle formation, and promoting more efficient lipid absorption [18,24]. Improved feed efficiency and nutrient utilization reduce compensatory lipid storage, effectively shifting energy partitioning toward productive growth rather than fat deposition, a phenomenon observed in broilers supplemented with multi-enzyme complexes in previous studies [25,26].

Papaya leaves themselves contain bioactive compounds, such as polyphenols and flavonoids, which have been reported to exert hypolipidemic effects by modulating hepatic cholesterol synthesis and lipid transport [1]. However, at higher inclusion levels, the elevated fiber content can limit lipid absorption, while excessive phytochemicals may impose metabolic stress, potentially offsetting some of the hypolipidemic benefits [27,28]. The present findings indicate that enzyme supplementation largely normalizes these effects, highlighting the critical role of structural carbohydrate degradation in stabilizing lipid metabolism in diets containing fibrous botanical ingredients like papaya leaf meal [29,30].

Enzyme supplementation also significantly enhanced endogenous antioxidant defense, reflected by increases in SOD, CAT, and total antioxidant capacity. Mechanistically, these improvements are likely attributable to multiple factors: enhanced nutrient availability, increased absorption of amino acids and micronutrients required for antioxidant enzyme synthesis, and reduced NSP-induced gut dysbiosis, collectively decreasing systemic oxidative stress [19,24,31]. Although papaya leaves contain antioxidant compounds such as polyphenols, flavonoids, and carotenoids, excessive inclusion (12%) increased MDA levels, indicating elevated lipid peroxidation, possibly due to higher fiber content and antinutritional factors that may impose metabolic stress at high inclusion levels [24,31]. These findings suggest a dose-dependent oxidative trade-off: while enzyme supplementation strengthens endogenous antioxidant defenses, high PLM levels may simultaneously elevate oxidative stress, likely due to increased structural carbohydrates and antinutritional compounds imposing additional metabolic burden. This pattern is consistent with previous reports on the effects of high inclusion levels of botanical feed ingredients in animals [17,32,33]. Importantly, enzyme supplementation partially mitigated these effects by improving fiber degradation and nutrient assimilation, although elevated MDA at 12% PLM underscores the need to carefully optimize papaya leaf meal inclusion to balance bioactive benefits with metabolic and oxidative safety [25,26,34].

Overall, these results support a mechanistic framework in which multi-enzyme supplementation alleviates fiber- and NSP-related limitations, optimizes lipid metabolism, and enhances antioxidant capacity in broilers fed fibrous botanical diets. Moderate inclusion levels of papaya leaf meal, in combination with targeted enzyme support, appear to maximize the functional benefits of phytogenic bioactives while minimizing metabolic and oxidative challenges, offering a sustainable strategy to improve broiler performance and health status [26,27,28].

The present study revealed that serum total protein, albumin, and globulin concentrations remained stable across all dietary treatments, suggesting that dietary PLM inclusion did not adversely affect protein metabolism or general physiological status. These results are consistent with previous reports showing that supplementation with papaya leaf meal at moderate levels does not compromise systemic protein status or humoral immunity in broilers [1,2,35]. Additionally, the absence of significant changes in ALT suggests that overt hepatocellular damage was unlikely. However, the significant increase in AST observed at the 12% PLM inclusion level may indicate increased metabolic load or mild physiological stress associated with higher dietary PLM levels. Elevated AST can reflect intensified hepatic metabolic activity or tissue turnover when animals are exposed to higher concentrations of structural carbohydrates and antinutritional compounds [1,16]. In the present study, the increased fiber content and presence of compounds such as tannins and saponins at the 12% PLM level may have imposed additional metabolic demands on the liver for detoxification and nutrient processing. These findings suggest that although papaya leaf bioactives may confer health benefits at moderate inclusion levels, excessive dietary PLM could impose subtle metabolic strain, highlighting the importance of optimized inclusion rates [16,17]. Collectively, the findings of this study, supported by previous experimental and meta-analytical evidence [2,3,4,6,35], provide a mechanistic framework for optimizing the use of papaya leaf meal in broiler diets. Multi-enzyme supplementation plays a pivotal role by degrading NSPs, reducing intestinal viscosity, enhancing nutrient release, and optimizing energy partitioning toward lean tissue growth [25]. Moderate PLM inclusion, around 6%, appears to strike an optimal balance, delivering functional bioactive compounds, such as polyphenols, flavonoids, and papain, without introducing excessive fiber or oxidative stress, thereby supporting growth, antioxidant defense, and metabolic homeostasis [25,36]. In contrast, high PLM inclusion (12%) increases fiber and antinutritional load, potentially inducing oxidative and metabolic challenges that may require additional interventions, including enhanced enzyme supplementation, pre-processing methods such as fermentation or acidification, or the inclusion of tannin-binding agents [35,36].

Therefore, the strategic integration of papaya leaf meal at moderate levels, combined with targeted enzyme supplementation, represents a practical and nutritionally sound approach to harness the phytogenic benefits of PLM while maintaining broiler health, growth performance, and metabolic stability. This framework provides a foundation for further optimization of botanical feed ingredients in commercial poultry production, balancing functional benefits with practical safety and efficiency considerations.

## 5. Conclusions

This study demonstrates that graded inclusion of papaya leaf meal (PLM) in broiler diets, combined with multi-enzyme supplementation, differentially influences growth performance, nutrient digestibility, carcass traits, lipid metabolism, antioxidant status, and serum biochemistry. Multi-enzyme supplementation consistently enhanced nutrient utilization, feed efficiency, lean tissue accretion, lipid profile, and endogenous antioxidant defenses, primarily by mitigating non-starch polysaccharide-related constraints and improving nutrient availability. Moderate PLM inclusion (≈6%) effectively delivered bioactive compounds that supported growth and oxidative balance synergistically with enzymes, without imposing excessive fiber or antinutritional stress. In contrast, high PLM inclusion (12%) increased metabolic demand and lipid peroxidation, highlighting the need for processing strategies or enhanced enzyme support at higher levels. Overall, these findings indicate that integrating PLM at moderate levels with targeted enzyme supplementation is a practical strategy to harness the functional benefits of papaya leaves while maintaining broiler health, metabolic homeostasis, and production efficiency.

## Figures and Tables

**Table 1:** Composition and analysis of experimental diets.

Ingredients %	Starter Diets			Grower Diets		
	Control	PLM 6%	PLM 12%	Control	PLM 6%	PLM 12%
Yellow corn	53.50	49.50	46.00	60.00	57.00	53.60
Soybean meal, CP 44%	32.00	29.50	26.80	24.00	21.40	18.50
Broilers concentrate, CP 48%	10.00	10.00	10.00	10.00	10.00	10.00
PLM *	0.00	6.00	12.00	0.00	6.00	12.00
Wheat bran	0.00	0.00	0.00	0.60	0.00	0.40
Vegetables Oil	3.10	3.60	3.80	4.00	4.20	4.10
Di-calcium phosphate	0.20	0.20	0.20	0.20	0.20	0.20
limestone	0.70	0.70	0.70	0.70	0.70	0.70
Common salt	0.25	0.25	0.25	0.25	0.25	0.25
Minerals & Vitamins ^a^	0.25	0.25	0.25	0.25	0.25	0.25
L-lysine	0.00	0.00	0.00	0.00	0.00	0.00
Total	100.00	100.00	100.00	100.00	100.00	100.00
Calculated analysis:						
ME (k cal/kg)	3043.65	3064.45	3053.98	3168.82	3175.58	3166.22
CP%	23.43	23.31	23.10	20.49	20.22	20.20
CF%	4.23	4.98	5.18	4.07	4.64	5.21
Ca%	1.01	1.02	1.00	0.99	1.00	1.00
Av. ph%	0.47	0.47	0.45	0.45	0.45	0.45
Lysine%	1.37	1.28	1.20	1.17	1.08	0.99
Methionine%	0.56	0.53	0.52	0.51	0.49	0.47
Cysteine%	0.38	0.36	0.33	0.33	0.32	0.30
Laboratory analysis:						
DM%	93.15	92.82	93.09	92.52	92.00	92.33
OM%	86.70	86.16	86.90	85.40	85.11	85.57
CP%	23.01	23.00	22.89	20.02	19.89	19.91
CF%	4.45	5.01	5.31	4.36	4.82	5.61
EE%	5.26	5.80	6.01	5.67	5.99	5.72
Ash%	6.45	6.66	6.89	7.12	7.49	7.76
NFE%	60.83	59.53	58.90	62.83	61.81	61.00
Moisture%	6.85	7.18	6.91	7.48	8.00	7.67

DM: dry matter, OM: organic matter, CP: crude protein, CF: crude fiber, EE: ether extract, NFE: nitrogen-free extract. * laboratory analysis of papaya leaf meal (PLM) was performed according to AOAC [9]: DM = 91.67%, Moisture = 8.32, Ash = 14.15%, OM = 77.53%, CP = 21.45%, CF = 14.26%, EE = 5.75%, NFE = 44.34%, ME kcal/kg = 2793.2. ^a^ each 1 kg Premix contained: Vit A 3,350,000 IU, Vit D3 760,000 IU, Vit E 6700 IU, Vit K3 335 mg, Vit B1 334 mg, Vit B2 1, 670 mg, Vit B6 500 mg, Vit B12 3.4 mg, Niacin 10,000 mg, Ca.D. Pantothenate 3334 mg, Biotin 16.7 mg, Folic acid 334 mg, Trace minerals: Iron 13,350 mg, Copper 3335 mg, Zinc 16,700 mg, Manganese 25,000 mg, Iodine 500 mg, Cobalt 84 mg, Selenium 100 mg, Additives: Ethoxyquin 600 mg, and Carrier (CaCo3) up to 1 kg.

**Table 2:** Effect of dietary papaya leaf meal with or without enzymes on growth performance of broiler chicks.

Items	Live Body Weight, g			Body Weight Gain, g			Feed Intake, g		
	1 d	21 d	42 d	1–21 d	22–42 d	1–42 d	1–21 d	22–42 d	1–42 d
PLM level									
Control	46.98	745.21	2272.77 ^b^	698.22	1533.82 ^a^	2232.04 ^b^	1180.04	3007.94	4187.98
6%	46.59	775.98	2327.88 ^a^	729.39	1551.89 ^a^	2283.86 ^a^	1186.02	3003.68	4183.45
12%	46.55	751.47	2218.87 ^c^	704.92	1467.40 ^b^	2182.32 ^c^	1193.93	2992.87	4181.81
Enzymes									
0 g/kg	47.23	708.88 ^b^	2213.56 ^b^	661.65 ^b^	1506.84	2178.86 ^b^	1167.04 ^b^	3027.87 ^a^	4177.42
0.5 g/kg	46.18	806.22 ^a^	2332.79 ^a^	760.04 ^a^	1526.57	2286.60 ^a^	1216.29 ^a^	2975.12 ^b^	4191.4
SEM	0.967	15.02	25.01	15.29	29.6	21.52	16.67	26.28	24.76
*p*-Value									
PLM	0.886	0.124	0.001	0.128	0.025	0.001	0.71	0.841	0.967
Enzymes	0.193	<0.001	<0.001	<0.001	0.473	<0.001	<0.001	0.024	0.498
PLM× Enzymes	0.665	0.106	0.205	0.123	0.344	0.074	0.546	0.595	0.319

^a,b,c^ treatment means in a column with different superscripts differ (*p* < 0.05). PLM: papaya leaf meal; PLM× Enzymes: interaction effect; SEM: Standard Error of Means.

**Table 3:** Effect of dietary papaya leaf meal with or without enzymes on feed, protein, and energy efficiency of broiler chicks.

Items	Feed Conversion Ratio			Protein Efficiency Ratio			Energy Efficiency Ratio		
	1–21 d	22–42 d	1–42 d	1–21 d	22–42 d	1–42 d	1–21 d	22–42 d	1–42 d
PLM level									
Control	1.700	1.970 ^ab^	1.880 ^ab^	2.520	2.200	2.290 ^ab^	0.190	0.170	0.180 ^a^
6%	1.630	1.920 ^b^	1.830 ^b^	2.630	2.210	2.330 ^a^	0.200	0.170	0.180 ^a^
12%	1.690	2.030 ^a^	1.920 ^a^	2.550	2.130	2.250 ^b^	0.190	0.160	0.170 ^b^
Enzymes									
0 g/kg	1.750 ^a^	2.020	1.920 ^a^	2.460 ^b^	2.160	2.240 ^b^	0.190 ^b^	0.170	0.170 ^b^
0.5 g/kg	1.600 ^b^	1.950	1.830 ^b^	2.690 ^a^	2.210	2.340 ^a^	0.200 ^a^	0.170	0.180 ^a^
SEM	0.041	0.045	0.022	0.061	0.048	0.036	0.004	0.003	0.002
*p*-Value									
PLM	0.217	0.087	0.007	0.168	0.142	0.069	0.141	0.087	0.026
Enzymes	0.001	0.078	0.001	0.001	0.279	0.003	0.001	0.615	0.001
PLM× Enzymes	0.386	0.510	0.444	0.356	0.131	0.170	0.166	0.064	0.249

^a,b^ treatment means in a column with different superscripts differ (*p* < 0.05). PLM: papaya leaf meal; PLM× Enzymes: interaction effect; SEM: Standard Error of Means.

**Table 4:** Effect of dietary papaya leaf meal with or without enzymes on the nutrient digestibility of broiler chicks.

Items	DM%	OM%	CP%	CF%	EE%	NFE%
PLM level						
Control	85.71	80.12	81.39	40.84	92.59	75.1
6%	87.43	79.51	81.07	35.63	92.51	74.82
12%	86.4	78.73	81.57	34.79	91.36	75.79
Enzymes						
0 g/kg	85.88	78.55	80.21	32.90 ^b^	91.39 ^b^	75.01
0.5 g/kg	87.15	79.7	82.48	41.27 ^a^	92.91 ^a^	75.47
SEM	0.953	0.96	1.46	3.23	0.866	1.74
*p*-Value						
PLM	0.216	0.217	0.943	0.157	0.305	0.85
Enzymes	0.119	0.166	0.074	0.005	0.046	0.745
PLM× Enzymes	0.014	0.061	0.454	0.013	0.112	0.909

^a,b^ treatment means in a column with different superscripts differ (*p* < 0.05). PLM: papaya leaf meal; PLM× Enzymes: interaction effect; SEM: Standard Error of Means; DM: dry matter; OM: organic matter; CP: crude protein; CF: crude fiber; EE: ether extract; NFE: nitrogen-free extract.

**Table 5:** Effect of dietary papaya leaf meal with or without enzymes on carcass yield of broiler chicks.

Items	Body Weight, g	Dressing%	Liver%	Gizzard%	Heart%	Fat%	Giblets%
PLM level							
Control	2348	78.43	2.130	1.770	0.51 ^b^	1.530	5.68 ^b^
6%	2313	77.09	2.250	1.860	0.64 ^ab^	1.690	6.74 ^a^
12%	2393	78.09	2.310	1.780	0.75 ^a^	1.350	6.24 ^ab^
Enzymes							
0 g/kg	2314	76.66 ^b^	2.320	1.870	0.720	1.660	6.55 ^a^
0.5 g/kg	2389	79.02 ^a^	2.140	1.730	0.540	1.390	5.89 ^b^
SEM	25.190	1.180	2.230	0.128	0.107	0.236	0.345
*p*-Value							
PLM	0.861	0.471	0.375	0.731	0.015	0.364	0.023
Enzymes	0.467	0.025	0.091	0.199	0.054	0.192	0.034
PLM× Enzymes	0.391	0.181	0.007	0.133	0.343	0.943	0.088

^a,b^ treatment means in a column with different superscripts differ (*p* < 0.05). PLM: papaya leaf meal; PLM× Enzymes: interaction effect; SEM: Standard Error of Means.

**Table 6:** Effect of dietary papaya leaf meal with or without enzyme mixture on serum biochemistry of broiler chicks.

Items	TP, g/dL	Alb, g/dL	Glob, g/dL	Glucose, mg/dL	ALT, U/L	AST, U/L
PLM level						
Control	5.61	1.65	3.96	200.87	20.25	20.37 ^b^
6%	5.43	1.53	3.9	199.25	19.75	20.25 ^b^
12%	5.67	1.41	4.25	213.5	23.37	27.30 ^a^
Enzymes						
0 g/kg	5.11	1.49	3.61	189.75	22.66	23.58
0.5 g/kg	6.03	1.57	4.46	219.33	19.58	21.67
SEM	0.755	0.369	0.862	24.11	1.85	1.86
*p*-Value						
PLM	0.944	0.806	0.907	0.813	0.135	0.001
Enzymes	0.151	0.804	0.234	0.15	0.057	0.225
PLM× Enzymes	0.662	0.509	0.952	0.773	0.514	0.303

^a,b^ treatment means in a column with different superscripts differ (*p* < 0.05). PLM: papaya leaf meal; PLM× Enzymes: interaction effect; SEM: Standard Error of Means; TP; total protein; Alb: albumin; Glob: globulin; ALT: alanine aminotransferase; AST: aspartate aminotransferase.

**Table 7:** Effect of dietary papaya leaf meal with or without enzymes on lipid profile of broiler chicks.

Items	TChol, mg/dL	TG, mg/dL	HDL, mg/dL	LDL, mg/dL
PLM level				
Control	172.00 ^b^	89.87 ^ab^	82.37	87.57
6%	207.13 ^a^	90.75 ^a^	95.5	79.77
12%	171.00 ^b^	79.87 ^b^	89.13	64.08
Enzymes				
0 g/kg	199.08 ^a^	92.16 ^a^	80.83 ^b^	98.91 ^a^
0.5 g/kg	167.66 ^b^	81.50 ^b^	97.16 ^a^	55.37 ^b^
SEM	13.34	4.8	9.1	15.89
*p*-Value				
PLM	0.022	0.066	0.374	0.343
Enzymes	0.009	0.014	0.031	0.003
PLM× Enzymes	0.215	0.874	0.254	0.554

^a,b^ treatment means in a column with different superscripts differ (*p* < 0.05). PLM: papaya leaf meal; PLM× Enzymes: interaction effect; SEM: Standard Error of Means. TChol: total cholesterol; TG: triglycerides; HDL: high-density lipoprotein; LDL: low-density lipoprotein.

**Table 8:** Effect of dietary papaya leaf meal with or without enzymes on the antioxidant profile of broiler chicks.

Items	TAC, U/mL	MDA, nmol/mL	SOD, U/mL	CAT, U/L
PLM level				
Control	1.160	63.00 ^b^	71.130 ^c^	75.13
6%	1.090	64.25 ^ab^	82.870 ^b^	82.00
12%	1.140	69.88 ^a^	89.380 ^a^	79.75
Enzymes				
0 g/kg	0.980 ^b^	60.58 ^b^	66.58 ^b^	70.25
0.5 g/kg	1.280 ^a^	70.83 ^a^	95.66 ^a^	87.67
SEM	0.202	2.691	2.796	4.242
*p*-Value				
PLM	0.943	0.045	<0.001	0.28
Enzymes	0.088	<0.001	<0.001	<0.001
PLM× Enzymes	0.982	0.410	0.001	0.019

^a,b,c^ treatment means in a column with different superscripts differ (*p* < 0.05). PLM: papaya leaf meal; PLM× Enzymes: interaction effect; SEM: Standard Error of Means. TAC: total antioxidant capacity; SOD: superoxide dismutase; MDA: malondialdehyde; CAT: catalase.

## Data Availability

The original contributions of this study are included in the article. Further inquiries can be directed to the corresponding author(s).

## Abbreviations

The following abbreviations are used in this manuscript:

PLM	Papaya leaf meal
NSP	Non-starch polysaccharides
SOD	Superoxide dismutase
CAT	Catalase
MDA	Malondialdehyde
ALT	Alanine aminotransferase
AST	Aspartate aminotransferase
HDL	High-density lipoprotein
LDL	Low-density lipoprotein
DM	Dry matter
CP	Crude protein
EE	Ether extract
FCR	Feed conversion ratio
